# Intestinal Epithelium in Inflammatory Bowel Disease

**DOI:** 10.3389/fmed.2014.00024

**Published:** 2014-08-25

**Authors:** Mehmet Coskun

**Affiliations:** ^1^Department of Gastroenterology, Medical Section, Herlev Hospital, University of Copenhagen, Herlev, Denmark

**Keywords:** barrier dysfunction, Crohn’s disease, inflammatory bowel disease, intestinal epithelium, tight junctions, ulcerative colitis

## Abstract

The intestinal epithelium has a strategic position as a protective physical barrier to luminal microbiota and actively contributes to the mucosal immune system. This barrier is mainly formed by a monolayer of specialized intestinal epithelial cells (IECs) that are crucial in maintaining intestinal homeostasis. Therefore, dysregulation within the epithelial layer can increase intestinal permeability, lead to abnormalities in interactions between IECs and immune cells in underlying lamina propria, and disturb the intestinal immune homeostasis, all of which are linked to the clinical disease course of inflammatory bowel disease (IBD). Understanding the role of the intestinal epithelium in IBD pathogenesis might contribute to an improved knowledge of the inflammatory processes and the identification of potential therapeutic targets.

## Introduction

**Figure 1:**
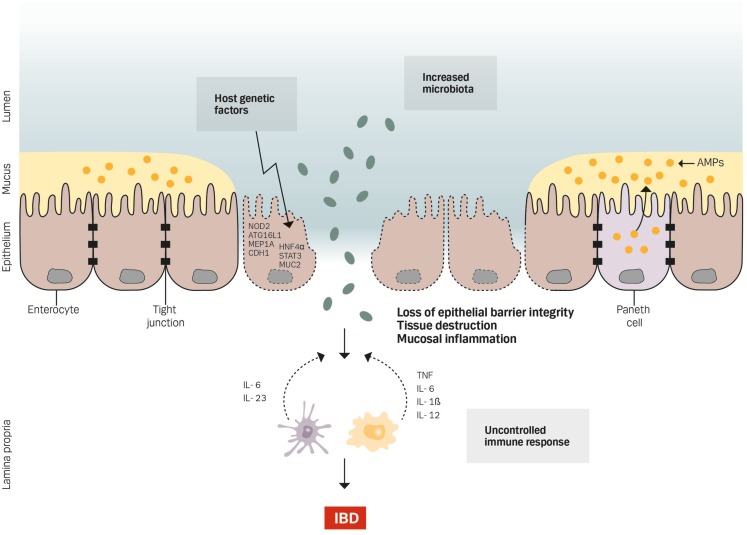
**Genetics, gut microbiota, and an uncontrolled immune response cause defects in epithelial barrier function by affecting the barrier integrity, increasing tissue destruction and mucosal inflammation**.

Inflammatory bowel disease (IBD) is characterized by a chronic idiopathic inflammation of the intestine and consists of two main forms, i.e., ulcerative colitis (UC) (1) and Crohn’s disease (CD) (2). The inflammation in CD can be transmural affecting any parts of the entire gastrointestinal tract, whereas UC is restricted to the mucosa of the colon. Although the etiology of IBD is largely unknown, it involves a complex interaction between genetic, luminal, and environmental factors that trigger an inappropriate mucosal immune response (Figure 1) (3–7). The importance of genetic susceptibility has over the past decades been established through genome-wide association studies, which have identified a total of 163 IBD-associated gene loci, most of which are associated with both CD and UC (110/163) – suggesting shared pathways in IBD pathogenesis despite differences in clinical phenotype – and 30 gene loci classified specifically for CD and 23 as UC specific (8). However, not all patients with genetic changes develop IBD. Therefore, other factors like environmental risk factors including diet, smoking, drugs, infections, geography, and stress have an important role in the pathogenesis of IBD (9). In particular, changes in the composition of the intestinal microbiota are likely the most important environmental factor in IBD by inducing an overactive immune response that harms the mucosal barrier (10–14). It is therefore not surprising that intestinal barrier dysfunction plays a key pathogenic role in IBD (15). Thus, IBD is a multifactorial disease driven by an exaggerated immune response to gut microbiota in a genetically susceptible host causing defects in epithelial barrier function and epithelial response to pathogens (Figure 1).

## Epithelial Homeostasis and Mucosal Inflammation

The intestinal epithelium forms the protective barrier and host defense against the harmful luminal microenvironment with selective permeability and absorption of nutrients. The epithelium is covered by a single-cell layer composed of different subtypes of specialized intestinal epithelial cells (IECs) including absorptive cells, goblet cells, enteroendocrine cells, Paneth cells, M cells, cup cells, and Tuft cells, all of which differentiate from epithelial stem cells (16–18). These subsets of IECs are functionally different and essential to maintain intestinal homeostasis by separating the intestinal lumen from the underlying lamina propria and by controlling the crosstalk between microbiota and subjacent immune cells. Thus, a dysregulation of the differentiation system for correct IEC formation has a crucial role in the pathogenesis of IBD (19). Indeed, several crucial genes for the differentiation of IECs have been demonstrated to become aberrantly expressed during IBD (20–22).

The epithelial monolayer is the main component of the epithelial barrier and its ability to act as a protective physical barrier is mediated by the formation of a web of tight junctions (TJs) that regulate the paracellular permeability and barrier integrity, production of mucus layer covering the luminal surface of the epithelium, and recognition of pathogens and production of antimicrobial peptides (AMPs) to ensure effective immunity (23). Hence, TJs seal the paracellular space between epithelial cells and separate the cell membrane into apical and basolateral domains, thus forming a physical barrier against foreign antigens. In fact, an altered expression and structural changes of the intestinal TJ proteins are closely associated with the development of IBD (24–26). Moreover, several pro-inflammatory cytokines, such as tumor necrosis factor (TNF)-α and interferon-γ, have been shown to increase TJ permeability and to induce apoptosis of IECs (27–29). This leads to the loss of epithelial barrier function and induces epithelial damage and ulcers that are present in mucosal inflammation. Indeed, a strong linkage has been established between abnormal intestinal permeability and mucosal inflammation in both CD and UC patients (30), which has been supported by several studies demonstrating a restored intestinal permeability in patients responding to anti-TNF therapy (31, 32). Moreover, among the various experimental mouse models of intestinal inflammation (33), two available models, namely, SAMP1/YitFc and C3H/HeJBir, develop spontaneous chronic inflammation due to defects in innate and adaptive immune responses. Thus, SAMP1/YitFc mice have epithelial dysfunction and increased permeability (34), whereas C3H/HeJBir mice are more likely to have dysregulated innate immune responses and bacterial clearance (35).

To support the epithelial barrier, a mucus layer covers the single layer of IECs (Figure 1), and the critical role of the mucus layer in microbiota sequestration has been demonstrated by animal studies with *Muc2*-deficient mice (36) – the producer of the main mucus protein secreted by goblet cells – and by reduced goblet cell numbers and depleted mucus secretion in IBD patients (37). In mice, *Muc2* deficiency results in a diminished mucus layer, elevated levels of pro-inflammatory cytokines, and development of spontaneous colitis. Moreover, the intestinal epithelial barrier is supported by the production of AMPs – mainly secreted by Paneth cells. These AMPs contribute to the mucus layer by preventing bacteria from reaching the epithelial surface or interact with the underlying immune system (Figure 1). Thus, the role of AMPs has been implicated in mucosal homeostasis and in the pathogenesis of several conditions including IBD (38–40). Indeed, a defective expression of AMPs has been revealed in patients with CD (41–43).

Apart from forming a tight protective barrier, IECs are actively involved in the innate immune response as many epithelial cells have pattern recognition receptors such as Toll-like receptors on the cell surface and nucleotide-binding oligomerization domain (NOD)-like receptors in the cytoplasm that are essential in sensing bacterial products and initiating the immune response through the pro-inflammatory transcription factor nuclear factor-κB to maintain homeostasis (18). Therefore, any defects in these IECs-related processes might trigger mucosal inflammation.

## Genetic Changes Affecting the Intestinal Epithelial Function

Genes within IBD-associated genetic loci highlight the importance of epithelial barrier defects (44). While the functional role of many loci or single-nucleotide polymorphisms is incompletely understood (8), several of the IBD-susceptibility genes (few examples are discussed below) are associated with different aspects of epithelial functions. Moreover, experimental mouse models of intestinal inflammation have been extensively used to investigate the importance of components of a healthy intestinal barrier function [reviewed in detail in Ref. (33)].

The nuclear transcription factor, hepatocyte nuclear factor 4α (HNF4α), encoded by *HNF4*α, regulates the expression of several components involved in epithelial TJs and intestinal permeability. HNF4α is down-regulated in patients with IBD and, moreover, mice with IEC-specific conditional knockout of *Hnf4*α are more susceptible to chemically induced colitis (20), indicating that HNF4α is crucial for the barrier function of the intestinal mucosa. Another example is the *CDH1* gene that encodes for E-cadherin, a cell adhesion molecule expressed in the epithelium, that is important for key morphogenetic processes such as cell growth, epithelial differentiation, and proliferation (45). Hence, loss or mislocalization of E-cadherin is involved in the pathogensis of IBD by increasing epithelial permeability (46).

Moreover, several polymorphisms in the *meprin 1A* (*MEP1A)* gene have been associated with UC (47). This gene encodes the α subunit of meprins found as a secreted form or as a membrane-bound form at the brush-border membrane in association with the transmembrane β subunit in IECs where their main function is to cleave diverse substrates such as laminins, TJ proteins, and cytokines (48–51). The expression of MEP1A is decreased in patients with active UC and experimental mice models with *Mep1A-*deficiency are more susceptible to chemically induced colitis (22, 52).

NOD2, encoded by *CARD15*, as the first genetic susceptibility locus for CD is an intracellular receptor that recognizes bacterial muramyl dipeptide and induces autophagy and bacterial clearance. NOD2 has, therefore, a central role in innate immune activation of epithelial cells attributed in part to Paneth cells. Autophagy is an important participant in the defense against intracellular invading pathogens and the NOD2-directed autophagy is dependent on, among others, the autophagy-related 16-like 1 (ATG16L1) protein, which is encoded by *ATG16L1* (53). Thus, carrying the CD-associated loss-of-function *NOD2* and/or *ATG16L1* risk variants displays an augmented inflammatory status (54) with a defective autophagy induction, bacterial clearance, and antigen presentation. As a consequence, the immune response is diminished, and luminal bacteria may invade the intestinal mucosa and trigger inflammation (55). However, neither partial nor complete loss of function of *Atg16l1* or *Nod2* leads to spontaneous intestinal inflammation (56, 57). Interestingly, specific deletion of the gene encoding X-box-binding protein 1 (Xbp1) – a transcription factor central to the unfolded protein response in the setting of endoplasmic reticulum (ER) stress – in IECs results in ER stress and Paneth cell impairment (58). Moreover, a recent study demonstrated that ER stress induced by IECs-specific *Xbp1* deletion was compensated by autophagy responses in Paneth cells. However, deletion of the autophagy-related gene, *Atg16l1* or Atg7, in addition to *Xbp1* developed severe spontaneous CD-like transmural ileitis (59). Thus, both ER stress and autophagy seem to be crucial regulatory mechanisms in intestinal homeostasis further underlying the importance of Paneth cells in intestinal inflammation, and this has indeed been supported by a recent study by Deuring et al. (60). In this study, the authors have shown that patients with CD, homozygous, or heterozygous for the *ATG16L1* risk allele, are associated with ER stress in Paneth cells (60).

A large number of mammalian cytokines modulate intracellular signaling by inducing the mitogen-activated protein kinase pathway (61), as well as the Janus kinase/signal transducer and activator of transcription (STAT) pathway (62). STATs are transcription factors that orchestrate an appropriate cellular response through target gene expression, and *STAT1*, *STAT3*, and *STAT4* have been reported as IBD-related susceptibility genes (8). In particular, animal studies have revealed the crucial role of an intact Stat3 in intestinal homeostasis and its protective role as IEC-specific *Stat3*-deficient mice are highly susceptible to chemically induced colitis (63).

Altogether, given the importance of many transcription factors, adhesion molecules, and immunological factors in the maintenance of the integrity of the epithelial barrier and regulating the homeostasis of epithelial cells, it is likely that subtle defects in epithelial gene function or expression may contribute to IBD pathogenesis.

## Concluding Remarks and Future Perspectives

The medical management of IBD includes glucocorticoids, immunomodulators (64), and anti-TNF-α biological agents (65, 66) as well as inhibitors of other molecular pathways (67). These drugs block key molecules that are involved in the induction and maintenance of inflammation in several signaling pathways, all of which have different contributions. However, no treatment strategy is curative or free of side effects. Hence, despite increased knowledge on its pathophysiology and improvements in medical therapy, about one-third of IBD patients need surgery due to an inadequate response or treatment failure to conventional therapy. Thus, achieving mucosal healing, preventing disease relapse, and avoiding complications are among the major goals in the management of patients with IBD (68–71). It is therefore likely that the mechanisms that drive mucosal inflammation differ between patients and that the inflammatory cascades are much more complex than initially thought.

Considering the culminating evidence that IECs are central to the maintenance of intestinal homeostasis and intestinal epithelium dysfunction is associated with IBD pathogenesis, potential IEC-based therapeutics will remain a crucial field of interest for IBD therapy. In fact, there are several indications that IECs are central in the response to TNF-α (72), and more importantly, IECs are a target of TNF-α inhibitors (73, 74).

Due to the complexity of signaling within the intestinal epithelium, monocultures of intestinal cells have until now limited their applicability as disease modeling. However, given the proper culture conditions, primary IEC cultures (organoids) can be maintained long-term *in vitro* and are therefore an invaluable new tool for research of more physiological relevance (75). Within the next several years, further studies and improved knowledge of the role of IECs in IBD will undoubtedly contribute to a better understanding of the pathogenesis of IBD, and will provide more insights into the potential and efficacy of IECs-based therapeutic opportunities and their application.

## Conflict of Interest Statement

The author declares that the research was conducted in the absence of any commercial or financial relationships that could be construed as a potential conflict of interest.
